# Supplementary material for: Local impacts on road networks and access to critical locations during extreme floods
Source: Sci Rep. 2022 Jan 28;12:1552. doi: 10.1038/s41598-022-04927-3 (PMC8799679; doi:10.1038/s41598-022-04927-3)
Supplement: Supplementary file 1 — Supplementary Information. [file 41598_2022_4927_MOESM1_ESM.pdf]

Supplementary Information for "Local impacts on road networks and access to critical locations during extreme floods"

Simone Loreti[1,2,*], Enrico Ser-Giacomi[3], Andreas Zischg[1,2], Margreth Keiler[1,2,4,5], and Marc Barthelemy[6,7,*]

[1]University of Bern, Institute of Geography, Bern, 3012, Switzerland
[2]University of Bern, Oeschger Centre for Climate Change Research, Mobiliar Lab for Natural Risks, Bern, 3012, Switzerland
[3]Massachusetts Institute of Technology, Department of Earth, Atmospheric and Planetary Sciences, Cambridge MA, 02139, United States
[4]University of Innsbruck, Department of Geography, Innsbruck, 6020, Austria
[5]Austrian Academy of Sciences, Institute of Interdisciplinary Mountain Research, Innsbruck, 6020, Austria
[6]Institut de Physique Théorique, CEA, CNRS-URA 2306, Gif-surYvette, F-91191, France
[7]Centre d'Analyse et de Mathématique Sociales (CNRS/EHESS), Paris, 75006, France
[*]To whom correspondence should be addressed. E-mail: simone.loreti@giub.unibe.ch or marc.barthelemy@ipht.fr

Supplementary Text

Generalized logistic function

We consider the generalised Verhulst's equation (1),

$$\frac{dF(t)}{dt} = \beta_1\left(F - C_1\right)\left(1 - \left(\frac{F - C_1}{D_1}\right)^{\gamma_1}\right) \tag{1}$$

where C_1 is the lower asymptote, D_1 is the upper asymptote, γ_1 is an exponent which allows to vary the shape of the (solution) sigmoidal curve and β_1 is the *intrinsic growth rate* (2) indicating the curve steepness. We then derive the corresponding generalised logistic function as follows:

$$\beta_1 \int_{t_0}^{t} dt = \int \frac{dF}{\left(F - C_1\right)\left(1 - \left(\frac{F-C_1}{D_1}\right)^{\gamma_1}\right)}$$

$$\beta_1(t - t_0) = ln\left|F - C_1\right| - \frac{ln\left|1 - \left(\frac{F-C_1}{D_1}\right)^{\gamma_1}\right|}{\gamma_1} + \text{const.}$$

$$\gamma_1\beta_1(t - t_0) = ln\left|F - C_1\right|^{\gamma_1} - ln\left|1 - \left(\frac{F - C_1}{D_1}\right)^{\gamma_1}\right| + \text{const.}$$

$$-\gamma_1\beta_1(t - t_0) = -ln\left|F - C_1\right|^{\gamma_1} + ln\left|1 - \left(\frac{F - C_1}{D_1}\right)^{\gamma_1}\right| + \text{const.}$$

$$-\text{const} - \gamma_1\beta_1(t - t_0) = ln\left|\frac{1 - \left(\frac{F-C_1}{D_1}\right)^{\gamma_1}}{(F - C_1)^{\gamma_1}}\right|$$

we use $W = \pm e^{-\text{const}}$,

$$
\begin{aligned}
We^{-\gamma_1\beta_1(t-t_0)} &= \frac{1-\left(\frac{F-C_1}{D_1}\right)^{\gamma_1}}{(F-C_1)^{\gamma_1}} \\
We^{-\gamma_1\beta_1(t-t_0)} &= \left(1-\left(\frac{F-C_1}{D_1}\right)^{\gamma_1}\right)\frac{1}{(F-C_1)^{\gamma_1}} \\
We^{-\gamma_1\beta_1(t-t_0)} &= \frac{1}{(F-C_1)^{\gamma_1}} - \frac{1}{D_1^{\gamma_1}} \\
\frac{1}{D_1^{\gamma_1}} + We^{-\gamma_1\beta_1(t-t_0)} &= \frac{1}{(F-C_1)^{\gamma_1}} \\
\frac{1+D_1^{\gamma_1}We^{-\gamma_1\beta_1(t-t_0)}}{D_1^{\gamma_1}} &= \frac{1}{(F-C_1)^{\gamma_1}}
\end{aligned}
$$

we use $A_1 = D_1^{\gamma_1} W$,

$$
\begin{aligned}
(F-C_1)^{\gamma_1} &= \frac{D_1^{\gamma_1}}{1+A_1e^{-\gamma_1\beta_1(t-t_0)}} \\
F-C_1 &= \frac{D_1}{\left(1+A_1e^{-\gamma_1\beta_1(t-t_0)}\right)^{1/\gamma_1}}
\end{aligned}
$$

Finally, we obtain the generalised logistic function:

$$
F(t) = \frac{D_1}{\left(1+A_1e^{-\beta_1\gamma_1(t-t_0)}\right)^{1/\gamma_1}} + C_1 \tag{2}
$$

With lower asymptote equal to $C_1 = 0$, Equation (2) would be solution of the Richards differential equation (3–6).

Supplementary Figures

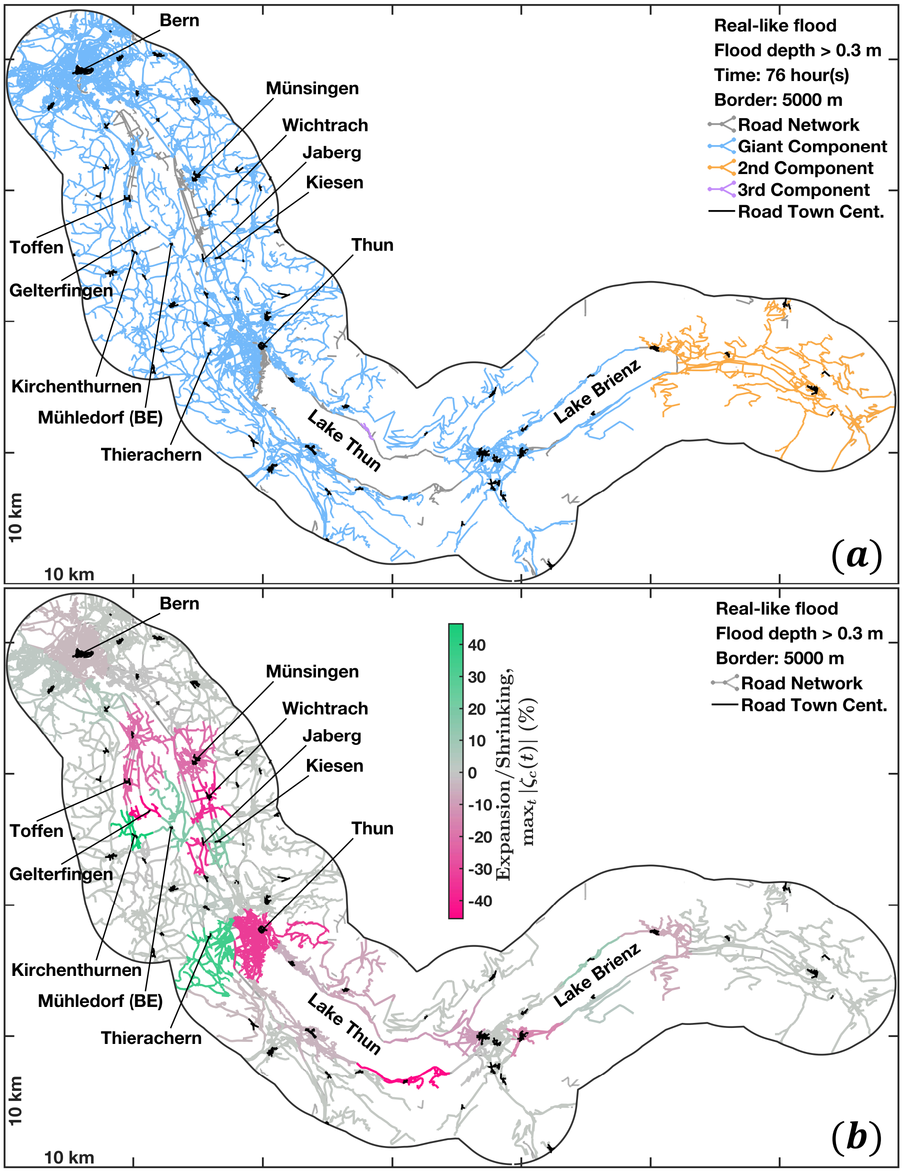

Supplementary Figure 1. A representative visual comparison between the percolation approach and our proposed framework and metrics. **(a)** Snapshot of the road network and of the three largest connected components during the maximum extension of a real-like flood (7), at $t_{MFE} = 76$ hours. The percolation approach shows that the mobility of people occurs within each of the clusters, but without the possibility of crossing from one cluster to another since they are disconnected. However, we do not have any information about the "internal" situation of each cluster. For example, it looks like that (apparently) vehicles can freely circulate without any obstacle or speed braking within the giant component. **(b)** Illustration of the road network, with highlight on the maximum value of $|\zeta_c|$ over the entire flooding period (for each town). With our approach, differently by percolation, we are able to infer the internal dynamics of each cluster, identifying the pivotal towns where large variations occur, both in space and in time. In particular, **(b)** shows the "re-routing mechanism" which corresponds to the mutual exchange of nodes among two or more adjacent towns (or groups of towns). We can clearly observe this mechanism between Thun and Thierachern, where Thierachern acquires some of the nodes lost by Thun, and between Kirchenthurnen and Gelterfingen, where Kirchenthurnen acquires some of the nodes lost by Gelterfingen. If we extend further our vision, we could observe the mechanism of mutual exchange of nodes among other adjacent towns, i.e. between the two groups of (i) Kirchenthurnen-Mühledorf and (ii) Gelterfingen-Wichtrach-Jaberg. Exception is made for the town of Münsingen, which just loses many nodes but there are not adjacent towns which acquire its lost nodes. This figure was produced with Matlab (8).

Supplementary Tables

	R^2	A	B	C	D	E	t_p	β	γ
$\frac{D_1}{\left(B_1+A_1e^{-E_1(t-t_p)}\right)^{1/\gamma_1}}+C_1$	0.9959	16.60	0.97	50.00	834.70	0.2295	52.2	0.145	1.581
$B_2+A_2e^{\beta_2 t}$	0.9975	3.73	35.89					0.0698	
$A_3e^{-\gamma_3 t}$	0.9981	2334							0.0134
$B_4+A_4e^{\beta_4 t}$	0.9800	64.94	416.30					0.0434	
$B_5+A_5e^{-\gamma_5 t}$	0.9911	3836	800.60						0.0158

Supplementary Table 1. Best fit values.

	Interurban Speed Limit $[km/h]$	Urban Speed Limit $[km/h]$
1 m road	-	50
2 m road	80	50
3 m road	80	50
4 m road	80	50
6 m road	80	50
8 m road	80	50
10 m road	80	50
entrance	50	-
exit	50	-
highway	100	-
motorways	120	-
service area	20	-
service areas connection	20	-
service area entrance	40	-
square	-	20

Supplementary Table 2. Speed limits adopted in this study for different road types and widths (9).

References

1. Verhulst, P.-F. Notice sur la loi que la population suit dans son accroissement. *Corresp. Math. Phys.* **10**, 113–126 (1838).
2. Tsoularis, A. & Wallace, J. Analysis of logistic growth models. *Math. Biosci.* **179**, 21–55, DOI: https://doi.org/10.1016/S0025-5564(02)00096-2 (2002).
3. Nelder, J. A. The fitting of a generalization of the logistic curve. *Biometrics* **17**, 89–110 (1961).
4. Richards, F. J. A flexible growth function for empirical use. *J. Exp. Bot.* **10**, 290–300 (1959).
5. Wang, X.-S., Wu, J. & Yang, Y. Richards model revisited: Validation by and application to infection dynamics. *J. Theor. Biol.* **313**, 12–19, DOI: https://doi.org/10.1016/j.jtbi.2012.07.024 (2012).
6. Turner, M. E., Bradley, E. L., Kirk, K. A. & Pruitt, K. M. A theory of growth. *Math. Biosci.* **29**, 367–373, DOI: https://doi.org/10.1016/0025-5564(76)90112-7 (1976).
7. Zischg, A. P. *et al.* Effects of variability in probable maximum precipitation patterns on flood losses. *Hydrol. Earth Syst. Sci.* **22**, 2759–2773 (2018).
8. MATLAB. *version 9.7.0.1216025 (R2019b) Update 1* (The MathWorks Inc., Natick, Massachusetts, 2019).
9. Finocchio, F. Fahrausweisbesitz in der schweiz seit 1950 (2004). Semesterarbeit.
